# Influence of Hearing Rehabilitation With Active Middle Ear and Bone Conduction Implants on Postural Control

**DOI:** 10.3389/fneur.2022.846999

**Published:** 2022-05-11

**Authors:** Ingmar Seiwerth, Antonia Brylok, René Schwesig, Torsten Rahne, Laura Fröhlich, Andreas Lauenroth, Timothy E. Hullar, Stefan K. Plontke

**Affiliations:** ^1^Department of Otorhinolaryngology, Head and Neck Surgery, Martin-Luther-University Halle-Wittenberg, Halle (Saale), Germany; ^2^Department of Orthopaedic and Trauma Surgery, Martin-Luther-University Halle-Wittenberg, Halle (Saale), Germany; ^3^Veterans Administration (VA) Portland National Center for Rehabilitative Auditory Research and Department of Otolaryngology-Head and Neck Surgery, Oregon Health and Science University, Portland, OR, United States

**Keywords:** transcutaneous hearing implant, active middle ear implant, bone conduction implants, postural stability, hearing amplification, balance

## Abstract

**Background:**

As audition also seems to contribute to balance control, additionally to visual, proprioceptive, and vestibular information, we hypothesize that hearing rehabilitation with active middle ear and bone conduction implants can influence postural control.

**Methods:**

In a prospective explorative study, the impact of hearing rehabilitation with active middle ear [Vibrant Soundbrige (VSB), MED-EL, Innsbruck, Austria] and bone conduction implants [Bonebridge (BB), MED-EL, Innsbruck, Austria] on postural control in adults was examined in three experiments. Vestibulospinal control was measured by cranio-corpography (CCG), trunk sway velocity (°/s) by the Standard Balance Deficit Test (SBDT), and postural stability with a force plate system, each time in best aided (BA) and unaided (UA) condition with frontal-noise presentation (Fastl noise, 65 dB SPL), followed by subjective evaluation, respectively.

**Results:**

In 26 subjects [age 55.0 ± 12.8 years; unilateral VSB/BB: *n* = 15; bilateral VSB/BB: *n* = 3, bimodal (VSB/BB + hearing aid): *n* = 8], CCG-analysis showed no difference between BA and UA conditions for the means of distance, angle of displacement, and angle of rotation, respectively. Trunk sway measurements revealed a relevant increase of sway in standing on foam (*p* = 0.01, *r* = 0.51) and a relevant sway reduction in walking (*p* = 0.026, *r* = 0.44, roll plane) in BA condition. Selective postural subsystem analysis revealed a relevant increase of the vestibular component in BA condition (*p* = 0.017, *r* = 0.47). As measured with the Interactive Balance System (IBS), 42% of the subjects improved stability (ST) in BA condition, 31% showed no difference, and 27% deteriorated, while no difference was seen in comparison of means. Subjectively, 4–7% of participants felt that noise improved their balance, 73–85% felt no difference, and 7–23% reported deterioration by noise. Furthermore, 46–50% reported a better task performance in BA condition; 35–46% felt no difference and 4–15% found the UA situation more helpful.

**Conclusions:**

Subjectively, approximately half of the participants reported a benefit in task performance in BA condition. Objectively, this could only be shown in one mobile SBDT-task. Subsystem analysis of trunk sway provided insights in multisensory reweighting mechanisms.

## Introduction

There is increasing evidence that hearing deterioration seems to be associated with reduced balanced control (1, 2). Considering the hypothesis that auditory cues may contribute to postural control—in addition to visual, proprioceptive and vestibular information, it can be assumed that hearing amplification with hearing devices not only serves as hearing rehabilitation but also as a stabilizing factor on postural control. This has been investigated several times in patients with hearing aids or cochlear implants (CI) (3–9), with most studies reporting a positive effect reported.

However, the exact mechanisms of interaction between auditory and vestibular, proprioceptive, and visual cues for maintaining postural control are still unknown. Although many studies with normal hearing and healthy subjects generally demonstrated a stabilizing effect (7, 10–14), some studies reported no influence (15–19) or even negative effects (16, 20, 21).

In patients with hearing impairment, the impact of hearing on balance control was so far studied mainly in patients with conventional hearing aids or with CI. When hearing rehabilitation with conventional hearing aids is not possible or insufficient in patients with conductive or mixed hearing loss, implantable active middle ear or bone conduction hearing devices offer an alternative. Since the effect of hearing amplification on postural control has not been investigated in these implantable devices until now, this study focuses on patients with hearing rehabilitation with a semi-implantable active middle ear [Vibrant Soundbrige (VSB), MED-EL, Innsbruck, Austria] or bone conduction [Bonebridge (BB), MED-EL, Innsbruck, Austria] hearing device. Here, conductive hearing loss is “bypassed” and sound is transmitted to the inner ear by bone conduction (22–24) or by coupling to middle ear ossicles or the round window (25, 26). Both devices consist of an external part, the audio processor, that is fixed by magnetic forces to the subcutaneous implant. The implantable part consists of a receiver coil, a demodulator, and an actuator—the floating mass transducer (FMT). In the VSB, the FMT can be attached to various middle ear structures like the long-incus process, the incus body, the stapes suprastructure or footplate or to the inner ear (the round window), while in the BB, the FMT is implanted in the temporal bone (25, 27, 28).

In the current literature, the methods of assessing balance or postural performance are inhomogeneous, which also illustrates the complexity of postural regulation processes. Considering different (patho) physiological pathways of balance subsystem interaction, different measurement methods are established for quantification of postural control.

The Unterberger (Fukuda) test, as an established assessment method for vestibulospinal control, is a frequently used method for clinical orientation of vestibular performance, offering objective measurement options as cranio-corpography (CCG) (29–31). For investigating the complex postural situation in activities of daily living, mobile trunk sway measuring systems based on the body's center of gravity, like the VertiGuard- System (32), are applicable.

An additional approach is the IBS-System (33, 34), a force plate system that quantifies different outcome parameters like postural stability and provides additionally insights into postural subsystem involvement by fast Fourier analysis of frequencies.

We hypothesize that hearing rehabilitation with active middle ear and bone conduction implants can influence postural control in this study. As exact interaction mechanisms are still unclear, different approaches of postural control quantification were used in this explorative study to find out which approach might be more relevant in a wide variety of patients. Additionally, subjective evaluation was included in the analysis.

## Materials and Methods

A prospective explorative study was conducted to examine the objective and subjective impact of hearing rehabilitation with active middle ear [Vibrant Soundbrige (VSB), MED-EL, Innsbruck, Austria] and bone conduction implants [Bonebridge (BB), MED-EL, Innsbruck, Austria] on postural regulation in adults. Inclusion criteria were hearing rehabilitation for at least 6 months, BMI of <35, and age between 18 and 75 years.

Before testing, microscopic otoscopy was conducted in all patients. For subjective balance evaluation, all patients were asked to answer the Dizziness Handicap Inventory (DHI) questionnaire. A video head impulse test (vHIT) of the horizontal semicircular canal was conducted. Additionally, directional hearing was tested in unaided (UA) and best aided (BA) condition. White noise was presented in random order at 65 dB SPL each from the angles 90, 45, 0, −45, and −90° with respect to the patient's position. Each angle was presented five times and the patients were asked to indicate the sound direction. The angle detection error was calculated as the mean square error.

In three experimental series, that were conducted on the same day, different balance measurement approaches were considered: Vestibulospinal control was measured by cranio-corpography (CMS10 measuring system for 3D motion analysis, Zebris Medical GmbH, Isny im Allgäu, Germany), trunk sway was measured in pitch and roll plane with the VertiGuard-System (Zeisberg, Metzingen, Germany) performing the Standard Balance Deficit Test (SBDT, 14 tasks), and postural stability was evaluated with a footplate-measurement system (neurodata GmbH, Vienna, Austria).

The experiments were all performed in a sound-insulated booth (DIN ISO 8253, reverberation time of <0.35 s, Fa. Industrial Acoustics Company GmbH, Niederkrüchten, Germany) with constant presentation of Fastl noise (35) at 0° and at 65 dB SPL in the free field. The speaker (Canton XL.3, Fa. Canton Elektronik GmbH & Co. KG, Weilrod, Germany) was adjusted each time to the individual ear position and was located at 1.85 m in front of the participants. In all subjects and all experiments, testing was conducted in BA and UA condition in pseudorandomized order.

Biometric consultation regarding statistical analyses was performed at the Institute of Medical Epidemiology, Biometry, and Informatics of the Martin-Luther-University Halle-Wittenberg, Germany. For statistical analysis, IBM SPSS Statistic software, version 28.0, for Windows (IBM, Armonk/NY, USA) was used. Testing for normality distribution was conducted by Kolmogorov-Smirnov test. Due to the descriptive character of this explorative study, there was no indication for multiple testing corrections. Depending on the respective experiment, the effect sizes *r* (small effect: ≥0.1, medium effect: ≥0.3, large effect: ≥0.5), *d* (small effect: ≥0.2, medium effect: ≥0.5, large effect: ≥0.8) (36), or respectively $\eta_{p}^{2}$ (≥0.1) (37) are indicators for the clinical relevance. Experiment-specific statistical analyses are further described in the respective sections. Graphs were prepared with Prism 9 software (GraphPad Software, Inc., La Jolla, CA, USA).

The study was approved by the responsible Ethics Committee (approval number: 2016-45) and conducted in accordance with the Declaration of Helsinki. All patients gave informed consent.

### Vestibulospinal Control

The subjects were asked to extend their arms with the palms facing the ceiling and to perform 50 test steps in place (Unterberger (Fukuda) stepping test) while they were wearing a blindfold. Changes in the participant's position and displacement in relation to the starting position were continuously recorded by CCG. Markers were located on the subject's right and left shoulder and at two points on the vertex (13) and their position was recorded by ultrasound (40 kHz, measuring rate 50 Hz). The two test conditions, BA and UA, were alternately tested, three times each. After completing the 50 test steps, the subject was, still blindfolded, led back to the starting position by the research supervisor on a continuously changing path. This was done to ensure that the test subject could not draw any conclusions about the previous change in position.

**Figure 1:**
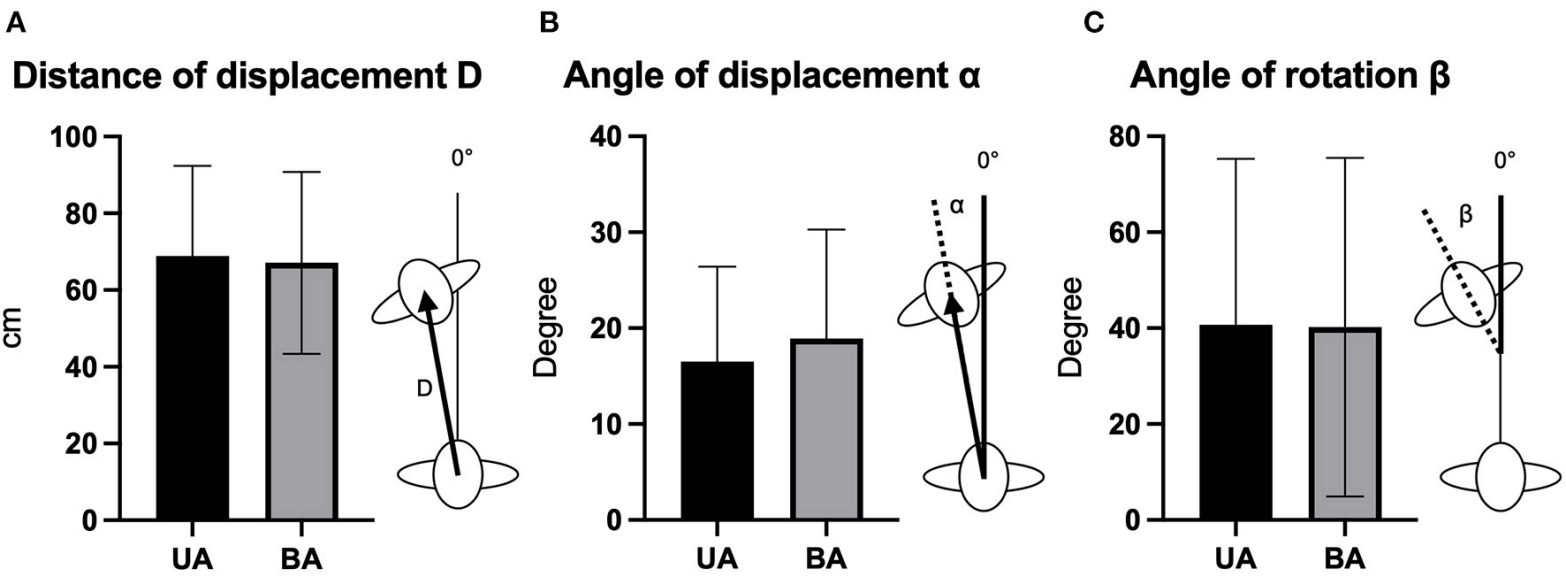
Comparative illustration and schematic depiction of the distance of displacement D **(A)**, the angle of displacement α **(B)**, and the angle of rotation β **(C)** for the conditions best aided (BA) and unaided (UA) (mean and standard deviation). cm, centimeter.

Measurement parameters were the distance of displacement D (cm) (the distance from the starting position after 50 steps), the angle of displacement α (°) (the angle between the distance line and the 0°-line) and the angle of rotation β (angle between the anterior-posterior body axis and the 0°-line) (Figure 1). Comparison of means was conducted by two-tailed Wilcoxon signed rank test.

### Trunk Sway Measurement

The VertiGuard-System, which consists of two orthogonally mounted gyroscopes, records body sway by measuring the angular velocity (°/s) at a sampling rate of 80 Hz and in pitch and roll dimensions. Patients wore the sensor close to the body's center of mass unit on their hips. Subjects performed 14 different tasks (Table 1) of the Standard Balance Deficit Test (SBDT) in pseudorandomized order in BA and UA condition. For selective analysis of the visual, proprioceptive, and vestibular subsystems, the SBDT tasks were categorized accordingly for further calculation: Based on the method described by Basta et al. (32) and modified to this study design, the values of a specific SBDT “basic” task were subtracted from the values of another SBDT task based on the “basic” task but with a disturbed visual, proprioceptive or vestibular input. For example, by subtracting the values of the task “standing on two legs with eyes open” from the task “standing on two legs with eyes closed,” net values for the visual component were obtained. The same procedure was done with all other SBDT tasks. Comparison of means was conducted by two-tailed Wilcoxon signed rank test.

**Table 1:** Trunk sway values measured with the VertiGuard-System in pitch and roll plane.

	**Pitch**				**Roll**			
	**°/s (mean** **±SD)**				**°/s (mean** **±SD)**			
**SBDT task**	**UA**	**BA**	***P***	***r***	**UA**	**BA**	***P***	***r***
standing on 2 legs eyes open	0.27 ± 0.10	0.28 ± 0.11	0.669	0.08	0.30 ± 0.12	0.31 ± 0.18	0.684	0.08
standing on 2 legs eyes closed	0.32 ± 0.13	0.32 ± 0.13	0.938	0.02	0.32 ± 0.14	0.34 ± 0.18	0.587	0.11
standing on 1 leg eyes open	2.03 ± 2.75	1.48 ± 1.40	0.348	0.18	2.97 ± 4.18	2.46 ± 3.02	0.205	0.25
standing on 1 leg eyes closed	3.67 ± 2.44	3.89 ± 2.76	0.112	0.31	6.39 ± 5.72	5.78 ± 4.63	0.855	0.04
walking 8 tandem steps eyes open	5.29 ± 1.62	5.29 ± 1.54	0.964	0.01	5.59 ± 1.45	5.59 ± 1.57	0.989	0.00
**standing on 2 legs on foam eyes open**	0.43 ± 0.20	0.42 ± 0.20	0.403	0.16	**0.46** **±0.24**	**0.56** **±0.32**	**0.01**	**0.51**
standing on 2 legs on foam eyes closed	0.76 ± 0.36	0.75 ± 0.31	0.752	0.06	0.82 ± 0.55	0.88 ± 0.53	0.374	0.17
standing on 2 leg on foam eyes open	3.21 ± 2.33	3.45 ± 2.77	0.55	0.12	5.16 ± 3.61	6.52 ± 5.69	0.112	0.31
walking 8 tandem steps on foam eyes open	5.41 ± 1.32	5.54 ± 1.51	0.741	0.06	7.39 ± 2.94	7.88 ± 2.54	0.288	0.21
**walking 3 m eyes open**	5.89 ± 2.05	5.58 ± 1.76	0.282	0.21	**7.3** **±3.05**	**6.11** **±1.72**	**0.026**	**0.44**
walking 3 m eyes open rotating head	5.88 ± 1.68	5.91 ± 1.83	0.904	0.24	7.64 ± 3.13	7.40 ± 2.13	0.919	0.02
walking 3 m eyes open pitching head	6.20 ± 1.83	6.19 ± 2.04	0.764	0.06	7.29 ± 3.14	7.00 ± 2.06	0.558	0.11
walking 3 m eyes closed	5.54 ± 1.97	5.46 ± 1.59	0.859	0.03	6.85 ± 2.86	6.40 ± 2.05	0.347	0.18
walking over barriers	15.6 ± 6.61	14.3 ± 5.57	0.144	0.29	15.7 ± 7.61	13.6 ± 6.81	0.08	0.34

*Clinically relevant results and tasks are marked in bold. BA, best aided; SD, standard deviation; SBDT, standard balance deficit test; UA, unaided*.

### Postural Stability

The Interactive Balance System (IBS, Neurodata GmbH, Vienna, Austria) consists of two force measurement plates with four sensors for forefoot and heel, respectively (sampling rate: 32 Hz). Subjects were standing without shoes on the plates while performing eight different tasks with changing sensory conditions (Table 2). Two foam pads were used depending on the respective tasks. Posturographic parameters were determined based on vertical pressure variation. A fast Fourier transformation was calculated for determining specific frequency band changes representing different postural subsystems (F1: visual and nigrostriatal, F2-4: peripheral vestibular, F5-6: somatosensory, and F7-8: cerebellar). IBS testing was also done for the UA and BA conditions in pseudorandomized order. Each of the eight exercises took about 32 s (Table 2). The stability indicator (ST) was determined as the root mean square of the differences between pressure distributions on the plates and describes the postural stability. The larger the stability indicator, the higher the instability of the person is to be rated. The weight distribution index calculates the standard deviation in the weight distribution on the plates assuming that 25% of the body weight is distributed evenly across the four plates. The Heel parameter describes the percentage load distribution between the forefoot and heel, whereas the left parameter describes the percentage load distribution between the left and right sides of the foot. Further detailed information of the IBS system and the frequency bands are provided in Bartels et al. (38), Friedrich et al. (39), Schwesig et al. (34), and Reinhardt et al. (40). Comparison of means between the UA and BA conditions was conducted by variance analysis (general linear model). Individual stability changes were evaluated by use of the quotient of ST values between the UA and BA condition.

**Table 2:** IBS task description.

**Stance position**	**Description**
NO	eyes open
NC	eyes closed
PO	eyes open, with foam pads
PC	eyes closed, with foam pads
HR	eyes closed, head rotated 45° to the right
HL	eyes closed, head rotated 45° to the left
HB	eyes closed, head up (dorso-flexed)
HF	eyes closed, head down (ventro-flexed)

### Subjective Analysis

After each experiment, the participants were asked to answer two questions: (1) How did, subjectively, the noise influence your sense of balance? Answer options were: “improved,” “no influence,” “deteriorated.” (2) Under which condition did you feel to achieve a better testing result? Answer options were “best aided,” “no difference,” or “unaided.”

## Results

### Demographics and Baseline Characteristics

**Figure 2:**
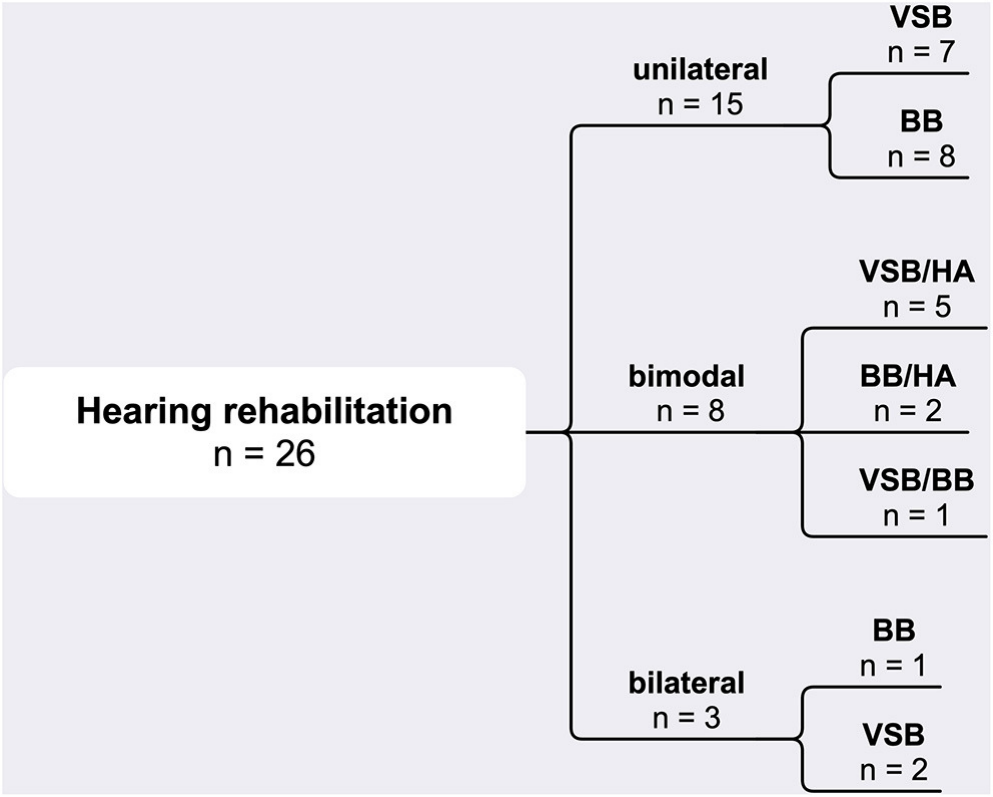
Overview of hearing rehabilitation modalities. BB, Bonebridge; HA, hearing aid; VSB, Vibrant Soundbridge.

Total 26 subjects were included in the study (m: *n* = 14, f: *n* = 12; age 55.0 ± 12.8 years). Out of these, fifteen patients had unilateral (VSB: *n* = 7, BB: *n* = 8); eight bimodal (VSB/HA: *n* = 5, BB/HA: *n* = 2, VSB/BB: *n* = 1), and three bilateral (VSB: *n* = 2, BB: *n* = 1) hearing rehabilitation (Figure 2). In the unilateral rehabilitated patients (*n* = 15), most of participants showed normal or near normal hearing (*n* = 9) on the contralateral side, but there was also moderate (*n* = 4), or severe hearing loss (*n* = 2).

In directional hearing testing, comparison of means showed an angle detection error of 42.0° (*SD* = 28.3) in BA and 46.2° (*SD* = 33.7) in UA condition (*p* = 0.46). The mean DHI-score was 17.1 (*SD* = 18.5). Testing of the vHIT (*n* = 22, in four subjects vHIT performance was not possible) showed mean gain-values of 1.01 (*SD* = 0.13) for the right and 0.91 (*SD* = 0.17) for the left side. In total, 13.6% of subjects (*n* = 3) showed unilateral corrective saccades with pathological gain value (<0.8) on the respective side.

### Vestibulospinal Control

Cranio-corpography analysis showed no relevant differences between BA and UA conditions in distance of displacement D, (BA: 67.1 cm, SD = 23.7; UA: 68.9 cm, SD=23.5; *p* = 0.24, *r* = 0.23), angle of displacement α (BA: 18.9°, SD = 11.4; UA: 16,5°, SD = 9.9; *p* = 0.19, *r* = 0.26) and angle of rotation β (BA: 40.2, SD = 35.3; UA: 40.7, SD = 34.6; *p* = 0.869, *r* = 0.03) (Figure 1).

### Trunk Sway Measurement With the VertiGuard-System

Trunk sway measurements of the SBDT tasks in roll plane showed a clinically relevant increase of sway in the BA condition in the task “Standing with 2 legs on foam with eyes open” (BA: 0.56°/s, SD = 0.32; UA: 0.46°/s, SD = 0.24, *p* = 0.01, *r* = 0.51) and a relevant sway reduction in BA condition in the task “walking 3 m forward with eyes open,” (BA: 6.11°/s, SD = 1.72; UA: 7.3°/s, SD = 3.05, *p* = 0.026, *r* = 0.44). No differences were seen in SBDT tasks in pitch plane (Table 1).

**Figure 3:**
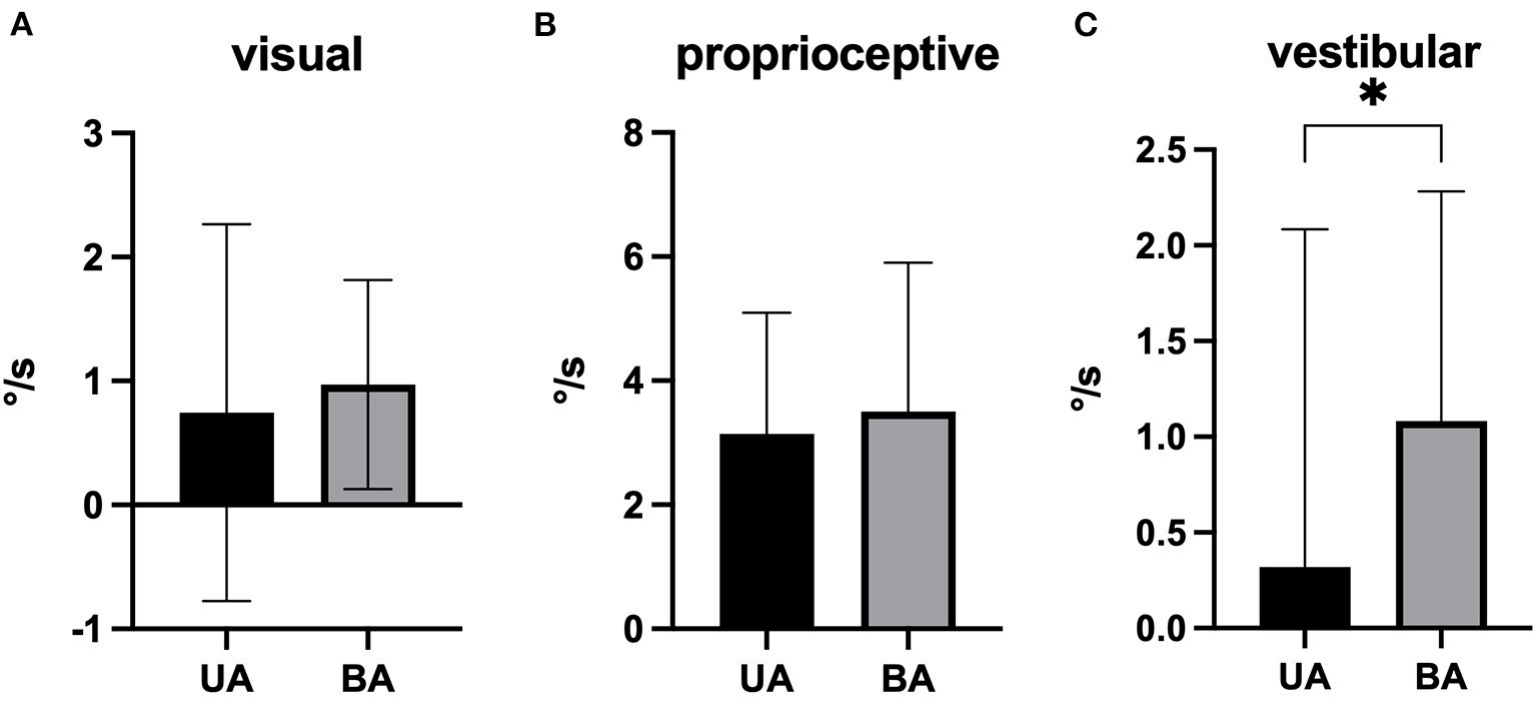
Mean net values of the trunk sway postural subsystem components in best aided (BA) and unaided (UA) condition [**(A)**: visual, **(B)**: proprioceptice, and **(C)**: vestibular] in roll plane (pitch plane not shown; mean and standard deviation). *p = 0.017.

Selective analysis of postural subsystem components (vestibular, visual, proprioceptive) revealed increased values for the vestibular component in BA condition (*p* = 0.017, *r* = 0.47), while visual and proprioceptive parts were not affected (Figure 3). No changes could be seen in pitch plane.

### IBS Force Plate Measurement

No clinically relevant differences were seen in the output parameters by comparison of means of the BA and UA test conditions (Table 3). Regarding individual changes in the stability-indicator (ST- parameter), 42 % of individuals improved stability in BA condition, 31% showed no difference, and 27% deteriorated (*n* = 25).

**Table 3:** Interactive Balance System (IBS)—Descriptive comparison (mean ± SD, *n* = 25) and analysis of variance for setting Bonebridge/Vibrant-Soundbridge best aided and unaided based on all positions (mean values).

	**Mean** **±SD**				
**Parameter**	**UA**	**BA**	***P***	** $\eta_{p}^{2}$ **	**d**
F1	19.1 ± 4.87	18.2 ± 5.12	0.386	0.032	0.18
F2-4	13.1 ± 4.50	12.8 ± 4.13	0.430	0.026	0.16
F5-6	6.05 ± 1.97	5.89 ± 1.57	0.415	0.028	0.17
F7-8	1.07 ± 0.42	1.04 ± 0.35	0.476	0.021	0.15
ST	33.9 ± 10.8	33.0 ± 10.1	0.276	0.049	0.23
WDI	5.55 ± 1.54	5.80 ± 1.59	0.264	0.052	0.23
Synch	522 ± 149	531 ± 123	0.624	0.010	0.10
Heel (%)	46.4 ± 7.31	45.8 ± 6.82	0.823	0.001	0.03
Left (%)	49.5 ± 3.03	49.6 ± 3.58	0.651	0.009	0.01

*F, frequency band; ST, stability indicator; WDI, weight distribution index; Synch, synchronization; Heel, Percentage of load distribution forefoot vs. hindfoot with description of heel loading; Left, Percentage of load distribution left vs. right with description of left side loading*.

### Subjective Estimation

Subjectively, 85% (CCG) and 73% (VertiGuard and IBS), respectively, felt that noise had no influence on their testing performance, 8% (CCG) and 4% (VertiGuard and IBS) felt an improvement and 8% (CCG), and 23% (Vertiguard and IBS) reported a deterioration.

**Figure 4:**
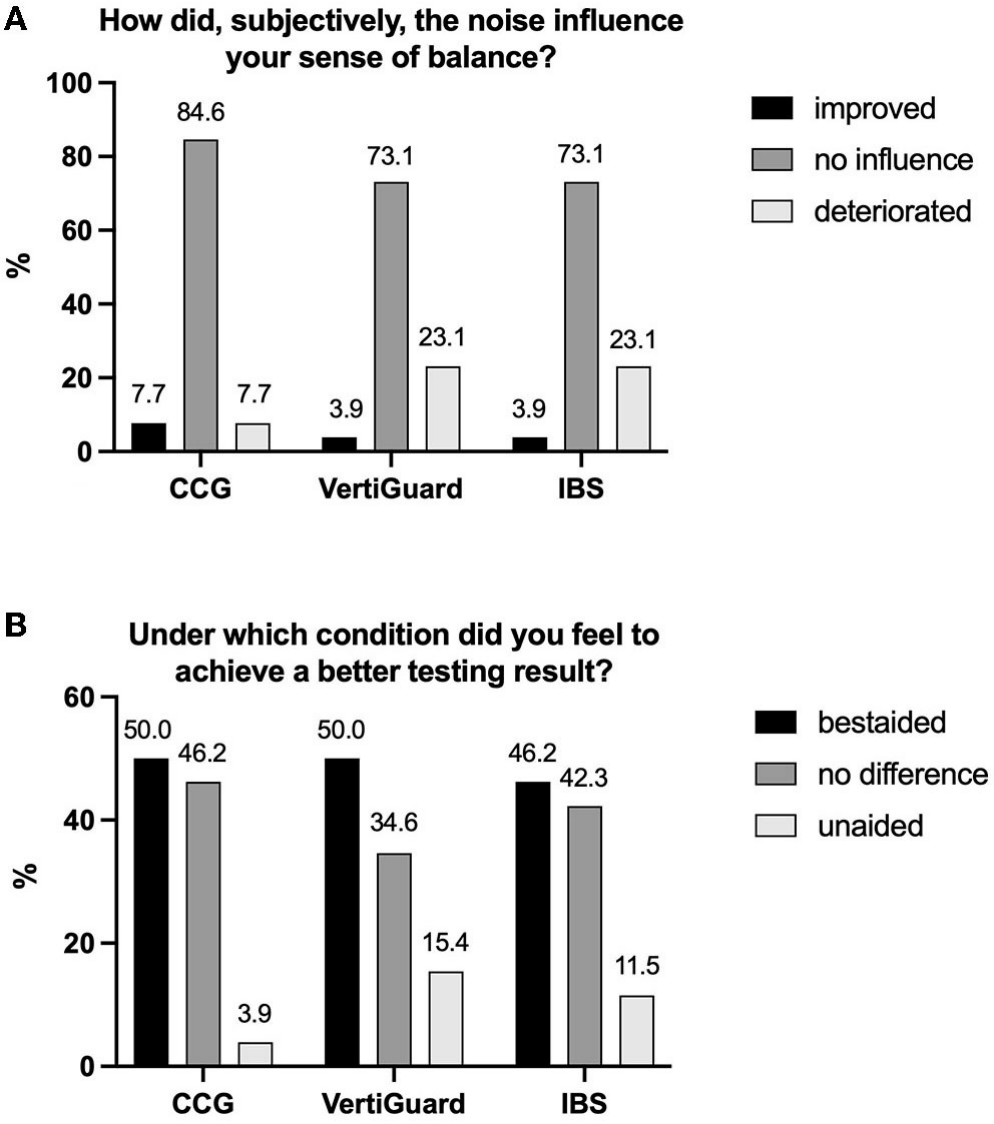
Descriptive illustration of the results of the two questions **(A,B)** that patients had to answers after each experiment. CCG, cranio-corpography; IBS, Interactive Balance System.

Regarding the influence of hearing rehabilitation, 50% (CCG and VertiGuard) and 46% (IBS) felt to have achieved a better test result in the BA condition, 46% (CCG), 35% (VertiGuard), and 42% (IBS) felt no difference and 4% (CCG), 15% (VertiGuard), and 12% (IBS) found the UA situation more helpful for test performance (Figure 4).

## Discussion

In the present study, the impact of hearing rehabilitation with active middle ear and bone conduction implants on postural control was evaluated considering different approaches of postural assessment.

In vestibulospinal control measurement, CCG-testing revealed no relevant differences in the performance of the Unterberger (Fukuda) stepping test between the UA and BA conditions regarding the distance of displacement, angle of displacement, and angel of rotation. A previous study demonstrated a clear benefit in normal hearing people, where a significant reduction of distance of displacement and angle of rotation were described in the condition with auditory input (13). A similar observation was also described in two other studies performing the Unterberger (Fukuda) test with sound presentation in normal hearing people (41, 42).

In the present study, the results of angle of rotation and angle of displacement were in both conditions generally found to be within the physiological range (30). However, the distance of displacement exceeded 50 cm, i.e., the physiological distance described by Fukuda et al. after 50 steps (29) in both conditions. This effect was also seen in a previous study with normal hearing subjects (13). A possible explanation could be the hypoechoic character of the testing room. Here, sound reflections from the side walls, which might serve as additional orientational cues, were not present, while in Fukuda (29), testing was conducted in a normal room. However, direct comparisons with the group of the present study are not possible, as the groups clearly differ in age and testing conditions. The normal healthy group from Seiwerth et al. (13) was tested with sound and without sound/earplugged, while in the present study, sound presentation was continuous and the conditions were best aided and unaided. However, in the present study, as Fastl-Noise (35) was presented continuously, residual unaided hearing may have still provided some auditory benefit. Patients could potentially have relied to the presented sound with their rest-hearing-ability, and as no deterioration was seen in the UA condition, even low noise levels might be sufficient for serving as supporting information for vestibulospinal control.

Although many studies analyzing the effect of audition on balance have used pressure plate measurements to assess postural control, a different, trunk sway-based approach was considered in the second experiment of our study: body sway was measured close to the center of body mass, including also mobile tasks, which is considered closer to daily-life conditions (43). As falls are relatively common events especially in older adults (44), mobile balance analysis should not be underestimated, especially regarding their clinical and practical relevance. In this study, from all 14 tasks of the SBDT, an effect could only be seen in two tasks in roll plane. No effects could be observed in pitch plane. In summary, in a stance task with additionally disturbed proprioception, hearing rehabilitation had a more disturbing effect on lateral sway, while in a mobile walking task, patients seemed to experience a benefit. In a study where gait analysis was conducted in three patients (5), an improvement in gait performance could be observed in a condition with hearing devices. Gait analysis was also done in a group of 12 bilateral CI-users and 13 bilateral hearing aid users (8). Here, no significant difference could be seen between aided and unaided conditions. However, some individuals improved in the aided conditions. Louza et al. (9) reported a small, but significant decrease of risk of fall in 33 adult CI patients with activated devices, which was more pronounced with additional presentation of music or speech text. Hallemans et al. (3) could demonstrate a positive influence of CI with music on gait performance in patients with bilateral vestibular areflexia. The subsystem analysis conducted in the trunk sway experiment showed relevantly increased values of the vestibular component in BA condition in roll plane, while no effect was seen in visual and proprioceptive disturbed tasks. This indicates that a higher use of this component was necessary for maintaining stable posture in BA condition compared to UA condition. This result may offer insights into audiovestibular interaction mechanisms. Only a few previous studies included the analysis of subsystem interaction so far. Maheu et al. (18, 45) described a sensory re-distribution as weighting increase of the visual component in healthy subjects (18) and a decrease of somatosensory dependence in patients with vestibular impairment on a force platform (45). In the trunk sway experiment of our study, effects can be seen only in roll plane but not in pitch plane. The underlying mechanisms remain unknown. However, the sound location, i.e., presentation from the front in contrast to lateral input, could have influenced the results. This could have served as a possible stabilizing effect, while the absence of lateral input, i.e., absence of wall reflections, was likely an uncommon situation for the patients. Raper and Soames (46) reported among others, that lateral sway seems to be more affected by pure tone input than anterioposterior values.

The third experiment, the investigation of postural stability with the force-plate-based IBS-System, revealed no difference in comparison of means of the BA and UA situation. Most force-plate based studies were done in patients with normal hearing, reporting generally a benefit of auditory input, while a few studies had their focus on patients with hearing aid rehabilitation. Neghaban et al. (47) reported a benefit of stability in the aided situation in 47 patients with bilateral hearing amplification, and Vitcovic et al. (7) observed a slight improvement in sway in 19 patients when they wore hearing aids with sound presentation. In a multicenter study with 69 adults (48), dynamic posturography was measured on a force plate in silence and with rotating sound. As results, no influence was seen in healthy subjects, a destabilizing effect of rotational sound was seen in patients with bilateral vestibulopathy and bilateral CI, and a stabilizing effect was seen in patients with unilateral CI. In another footplate-based study (49), CI-users (*n* = 8) deviated laterally when their eyes were closed while sound presentation could induce an improvement of that abnormal deviation. No effects were seen in healthy people (*n* = 8). A further study analyzing the effect of bilateral hearing aid use on balance, where posturography was measured on a force plate in 22 adults, revealed no significant benefit of hearing aids on balance (50).

Apart from providing general posturographic parameters like stability, the IBS-System allows a differentiated analysis of postural regulation subcomponents. Here, no differences could be seen in the specific frequency bands between the conditions. In a previous study based on the same IBS-System but with normal hearing subjects, a downregulation of the frequency bands F1 (visual and nigrostriatal) and F2-4 (peripheral-vestibular) was reported in sense of a reweighting of postural subsystems in the presence of sound (51). This effect could not be observed in our study. Regarding the 42% patients who improved stability in our study in BA condition, it would be interesting to find out why some patients improved, and others did not. However, no pattern of stability improving factors could be observed in this study population.

Regarding the subjective evaluation, there was a partially considerable discrepancy between the answers to the two questions as well as between the objective evaluation and the subjective estimation depending on the respective task. Only 4–7% of participants felt that noise improved their balance and 7–23% reported even deterioration by noise. On the other hand, nearly half of subjects reported to have better performed the task in the BA condition, while only 4–15% found the UA situation more helpful for test performance. In summary, hearing rehabilitation itself was seen subjectively as a benefit by a larger group of patients, while the presented sound itself seemed to be more a disturbing factor during the experiments. In this study, Fastl noise (35), a white noise with unpredictable interruptions, was presented with the aim to avoid possible noise reduction effects of the audio processor of the hearing device and not to excite too much cognitive attraction. However, the sound seems to have been perceived as unpleasant by the subjects.

That uncomfortable noise can deteriorate balance was shown by Chen et al. (16) where increased sway on a footplate was reported in the presence of unpleasant noises. Louza et al. (9) reported that in a study with CI-patients, music led to a reduction of risk of falls, while white noise showed no significant improvement.

To our knowledge, this is the first study analyzing the impact of hearing rehabilitation with active middle ear and bone conduction implants on postural control. While CI surgery can be associated with postoperative vertigo (52, 53), vestibular pathology is not expected as complication of BB surgery. However, there may be a potential effect of transcranial stimulation by bone conduction, also considering a stimulation of the contralateral side.

In VSB surgery, even decreases of bone conduction hearing thresholds were reported as postoperative complications (28), vestibular loss is not a prominent complication. Theoretically, it is conceivable that in patients with semicircular canal dehiscence syndrome (54), bone transmitted sound can induce vertigo, or that in cases of accidental intracochlear lesions in RW coupling in VSB surgery, patients show vestibular loss. However, this was not reported in the patients of this study.

Limitations of this study include its descriptive character as well as a large spread of vestibular function of the study group, as can be seen in the DHI results (DHI mean score of 17.1, *SD* = 18.5). As the focus of the present paper was on the effect of hearing rehabilitation with active middle ear and bone conduction implants across a wide variety of patients, subjects with possible sensory impairment were not excluded from the study. However, to answer the question how vestibular impairment is affected by auditory input, further specific work is necessary including more detailed vestibular diagnostic. Furthermore, the inhomogeneity of hearing rehabilitation in the study group (Figure 2) could be also a reason for the reduced sound localization ability values with an angle detection error of 42.0 in BA and 46.2° in the UA condition, compared to 0.6° in normal hearing people (13). As the study design was with frontal presentation of noise and without sound reflection ability due to the hypoechoic testing room, sound localization ability could play an important role. Anton et al. (55) concluded in a gait study in healthy subjects, that localization ability of auditory signals could improve postural control. The audiological and vestibular inhomogeneity of the study population may also be a reason for the individual differences of the motoric output in the different experiments. Also here, more research remains to be done to figure out why some people may have a benefit and others not.

## Conclusion

From this exploratory study, we conclude that hearing rehabilitation with active middle ear and bone conduction implants had a subjectively positive effect on postural control on approximately half of participants. Noise quality seemed to play an important role, as the presented noise subjectively showed a rather indifferent or destabilizing effect. Objectively, an improvement could be shown only in a walking-task in trunk sway measurement and in individual stability changes on a force plate measurement. Subsystem component analysis of trunk sway revealed a higher affection of the vestibular component auditory input, providing insights into audiovestibular interaction pathways.

## Data Availability Statement

The raw data supporting the conclusions of this article will be made available by the authors, without undue reservation.

## Ethics Statement

The studies involving human participants were reviewed and approved by the Ethics Committee, Medical Faculty, Martin-Luther-University Halle-Wittenberg. The patients/participants provided their written informed consent to participate in this study.

## Author Contributions

IS, SP, TR, RS, and TH contributed to the designed the study. IS and AB performed the scientific investigations and data analysis and drafted the manuscript. All authors reviewed, commented, and approved the final version of the manuscript.

## Funding

The study was done solely with intramural resources of the Department of Otorhinolaryngology, Head and Neck Surgery, Martin-Luther-University Halle-Wittenberg, Halle (Saale), Germany.

## Conflict of Interest

SP, TR, and LF are investigators in controlled post market entry studies or investigator-initiated research projects with implantable active middle ear and bone conduction hearing devices produced by MED-EL, Innsbruck, Austria. SP is investigator in research projects with grant support to the department of the corresponding author. SP received honorary for lectures from MED-EL Austria and MED-EL Germany. None of this was related to the research presented here. This study was not funded by MED-EL Austria or MED-EL Germany. There are not other commercial or financial relationships that could be construed as a potential conflict of interest. The remaining authors declare that the research was conducted in the absence of any commercial or financial relationships that could be construed as a potential conflict of interest.

## Publisher's Note

All claims expressed in this article are solely those of the authors and do not necessarily represent those of their affiliated organizations, or those of the publisher, the editors and the reviewers. Any product that may be evaluated in this article, or claim that may be made by its manufacturer, is not guaranteed or endorsed by the publisher.
